# Buffalo Medical Association

**Published:** 1860-03

**Authors:** Wm. H. Butler

**Affiliations:** Secretary


*Proceedings of the Buffalo Medical Association.* January Meeting, reported by Wm. H. Butler, M.D., Secretary.

Dr. Hutchins reported the following case of *Rupture of the Uterus:* As Dr. Gould is absent, to whom it properly belongs, I will mention a case of rupture of the uterus, which occurred a few days since.

Dr. Gould was called early in the morning, and found a woman about twenty-five years old, the mother of two children, in labor with her third child. There had been no motion for some days. The arm presented. A German woman, who had acted as midwife, had drawn violently on the arm for three or four hours. Dr. Gould immediately attempted to turn, but was unable to do so. He came and requested me to go and assist him. On reaching the patient I also made an attempt to turn, but from the violent contraction of the uterus could not succeed. As I supposed it impossible for any person to turn with such a state of things, I requested Dr. Gould to hand me the perforator which I had carried with me, my hand still being in the vagina, that I might commence the dissection and removal of the fœtus. He said that he would first prefer a trial by a third person, and left, to get some one else to make the effort. I remained with the woman during his absence, which was about half an hour; soon after he left the woman got on her knees by the side of the bed, and had several very violent pains. Just before his return she got into bed again. Dr. Miner came back with Dr. Gould, and immediately introduced his hand, and turned without any difficulty. After the removal of the fœtus, which had been dead for some time, Dr. Miner introduced his hand into the uterus, having been surprised at the want of pains during the delivery of the fœtus, and discovered that there was a rupture of the uterus. He passed his hand up into the cavity of the abdomen. After he had withdrawn his hand I introduced mine; found the rupture, and withdrew the placenta. The woman died three days after.

Dr. Eastman remarked that he had noticed that most of these cases had occurred where a midwife had been in attendance. He had been called in on two occasions where midwives had been first employed, and after difficulty was encountered, a doctor was sent for. One case he was called to see, a few days ago, he found a midwife making violent traction on the arm, which had presented. In this case, owing to the length of time in labor, he had great difficulty in turning the child, but finally did it to the great relief of the woman.

Dr. Cronyn had been called to a case where a midwife had preceded him, and found that she, finding a convenient handle protruding—the arm—had pulled on it so hard that it only held by the skin.

Dr. Cronyn also reported a case of labor where the child, after birth, continued to vomit blood. The presentation was the right knee; the doctor pushed the knee gently back, and after a few pains the breech presented, and the woman was rapidly delivered of a healthy female child, nearly asphyxiated, but restored by cold water. The second day after he was requested to see the child, which had been vomiting blood, and continued to vomit when anything was drank for thirty hours, or until death ensued. Every remedy was tried that was thought advisable, in vain. There were no symptoms of cyanosis; the heart and lungs seemed healthy; no cough. The doctor thinks that there must have been at least a pint of blood thrown up during the time the child lived. The case related was rare to Dr. C., and he asked if members had met with cases like it. A post-mortem could not be obtained.

Dr. Butler stated in relation to the case of rupture of the uterus, reported by Dr. Eastman, that a year ago he had been requested to make a post-mortem, where a female had died suddenly after turning and delivery. In this case also a midwife had been in attendance for the greater part of the night, and finally sent for a doctor, who turned and delivered. The patient sank, and died a few hours after.

The case was investigated, and on making a post-mortem, Dr. B. found rupture of the uterus upon the right side.

Dr. Butler related the particulars of a post-mortem he had made at the request of Coroner Randall. The subject was a woman about twenty-seven years old, whose person bore evidences of secondary eruption, with the whole body and intestines loaded with fat. The coats of the stomach were highly inflamed, softened, and it had an alcoholic smell. There were two wounds, as if caused by a knife; one across the lower part of the back, the other between the eighth and ninth ribs of the right side, running obliquely upward into the cavity of the chest. Both wounds had partially glued together, but the finger readily entered the thoracic cavity through the upper wound; and around the internal edge of the cut a rim of plastic lymph was effused. The lung on this side was entirely gone, save a very thin shred close to the mediastinum; and in its place was a mass of thick pus, which was dipped out with a cup. Not even a bronchial tube remained.

He reported the case for the purpose of asking the opinion of members of the Association as to the probability of the entire disorganization of the lung in a person of bad habits, with the fatty degeneration present in this case, in sixteen days. (It appeared in evidence afterwards that the woman lived sixteen days after being stabbed.) The case was interesting in a medico-legal point of view, and this question was in reality of considerable importance in its effect on the accused.

Dr. Cronyn had never seen a case precisely like the one related, but saw a patient who was admitted into the Toronto Hospital, and who died a few days after admission. On examination, after death, it was found that the lung on the right side was entirely disorganized and gone. This showed, at least, that a person might live with one lung entirely gone.

